# The bed nucleus of the stria terminalis in threat detection: task choice and rodent experience

**DOI:** 10.1042/ETLS20220002

**Published:** 2022-11-23

**Authors:** Emily R. Sherman, Jasmine J. Thomas, Emma N. Cahill

**Affiliations:** 1 Department of Physiology, Development and Neuroscience, University of Cambridge, Bristol CB2 3EB, U.K.; 2 School of Physiology, Pharmacology and Neuroscience, University of Bristol, Bristol BS8 1TD, U.K.

**Keywords:** anxiety, bed nucleus stria terminalis, behaviour, fear, mouse, rat

## Abstract

Behavioural reactivity to potential threat is used to experimentally refine models of anxiety symptoms in rodents. We present a short review of the literature tying the most commonly used tasks to model anxiety symptoms to functional recruitment of bed nucleus of the stria terminalis circuits (BNST). Using a review of studies that investigated the role of the BNST in anxiety-like behaviour in rodents, we flag the certain challenges for the field. These stem from inconsistent methods of reporting the neuroanatomical BNST subregions and the interpretations of specific behaviour across a wide variety of tasks as ‘anxiety-like’. Finally, to assist in interpretation of the findings, we discuss the potential interactions between typically used ‘anxiety’ tasks of innate behaviour that are potentially modulated by the social and individual experience of the animal.

## Difficulties with definitions: anxiety and the bed nucleus of the stria terminalis

Some concepts of emotional experience used in the neuroscientific literature are both intuitive but nonetheless difficult to experimentally define, including stress, fear and notably anxiety. What are diagnosed as anxiety disorders have fluctuated over time, remarkably in light of what neurobiological experimental evidence has demonstrated to be associated with particular symptoms that characterise specific spectra of disorders of anxiety, such as trauma, stressor-related or obsessive-compulsive disorders [1]. Attempts to map particular brain systems to a rodent behavioural readout that could model symptoms of anxiety have been met with many challenges in terms of establishing a predictive theoretical framework [2,3]. A practical distinction for rodent experimental design between fear and anxiety driven defensive behaviour (discussed below) can be made from the nature of the physical external trigger. Fear is argued to be triggered by perception of an obvious or imminent threat, whereas anxiety is thought to represent the anticipation of an ambiguous or distant threat [4–6]. The duration of exposure to the threat has also been used to define brief presentations as fear-eliciting, whereas sustained presentations would elicit anxiety [7]. Clinical studies have also used the threat imminence, or duration, as protocol parameters to refine what brain regions become recruited by anxiety rather than fear [7,8]. One such area that did not prominently feature in the earlier neuropsychological models of anxiety is a region known as the Bed Nucleus — or Nuclei, given the complex collection of substructures — of the Stria Terminalis (BNST).

Studies indicated that the Bed Nuclei of the Stria Terminalis (BNST) may be preferentially recruited over the amygdala nuclei for anxiety-evoking rather than fear-evoking stimuli [9–11]. Both associative aversive learning protocols [8], and stimuli that evoke ‘innate’ anxiety such as phobic images of spiders have been used to investigate recruitment of BNST activity in humans [12]. Despite the attraction of a brain region being more strongly associated with responses defined as anxiety-like, there remains debate about how real a biological difference there is in the circuitry underlying what we define operationally as fear or anxiety [13]. Herein, we focus the review on studies using mice or rats that explore targeting the BNST with a behavioural readout that is argued to model symptoms of anxiety.

In laboratory rodents, the BNST is posited to have a role in innate responses to a stressor, such as brightly lit environment or predator odour [14–16] and in cases where the stressor potentiates subsequent startle responses [17,18]. Nonetheless, evidence for a role for BNST in the encoding of a conditioned experience is growing [19,20]. The nature of cue — discrete or contextual — used to trigger either conditioned freezing or flight behaviour was initially thought to control the involvement of the BNST, however the refinement of the task protocols by modulation of cue predictability, as well as its physical properties, has provided a more nuanced interpretation of when the BNST contributes to anxiety-related behaviour [21–24].

**Figure 1.**
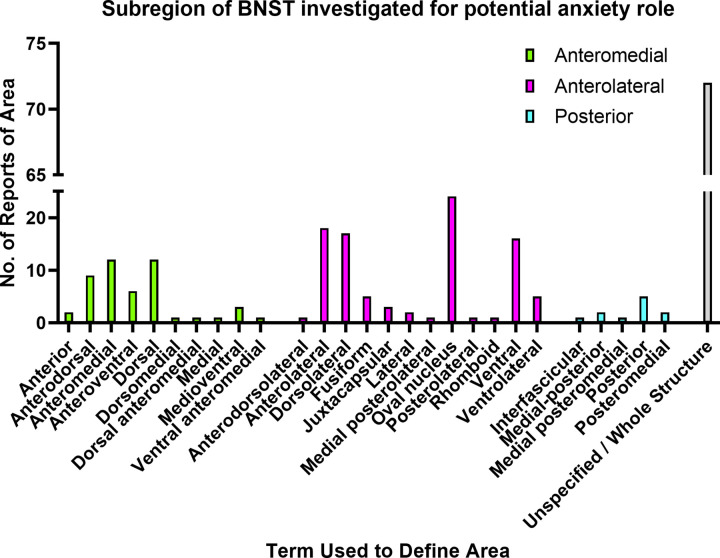
A representation of the anatomical focus of the rodent literature with regards to reporting a role of the BNST in anxiety-like behaviour. The vast majority (31.7%) of papers do not specify a particular region (‘Unspecified’), referring to the BNST as a whole structure. Note that this representation may refer to the same anatomical area with different terminology used in the study. Subregions are grouped by the categorisation of Dong and Swanson [27] to aid standardisation.

A significant challenge remains the limited resolution of the subnuclei of the BNST in human functional magnetic resonance imaging [9]. The BNST is a limbic forebrain structure, found encapsulating the anterior commissure, which is considered to be part of the so-called ‘extended amygdala’ and is well connected to form part of a threat detection system [25,26]. We will not review the details of the chemoarchitecture of the BNST subnuclei herein, for which we direct the reader to thorough reviews on the topic [7,25,26]. Despite progress on refining the connectivity of the BNST subnuclei, a lack of convention in terminology for naming subregions limits comparison across interventional studies [26,27] (see Figure 1). Moreover, there is evidence in the literature that the BNST may be sexually dimorphic (with functional consequences) both in rats [28,29], mice [15,30,31] and in humans [32,33]. It should be noted also that resources like the neuroanatomical atlases commonly used for reference and reporting, such as those published by Paxinos, Watson & Calabrese (first edition 1982), were generated using male animals.

To illustrate the array of terms that were used to describe the anatomical subdivisions of the BNST and the variety of tasks used to investigate anxiety mechanisms, we performed a systematic literature review of recent rodent studies. Searches were conducted using PubMed, the Cochrane Library and pre-print archives (BioRxiv, MedRxiv, PscyArXiv) using the Boolean operators:

“*(((BNST) OR (Bed nucleus stria terminalis)) AND ((anxiety) OR (‘sustained fear’)) AND ((‘rodent’) OR (‘mice’) OR (‘rat’))”*

This generated 584 unique literature records (published from 1997–2021). Pre-prints were included as the purpose was to sample what terms and tasks are being actively used in recent studies of the BNST. Two authors screened the papers with exclusion criteria that articles should be open access, in English, and have investigated a functional role of the BNST in tasks proposed to model anxiety-like behaviour (see Supplementary Figure S1). A total of 173 records were deemed appropriate for inclusion based on their relevance to anxiety-like behaviour and the BNST (to cut off date of 28th of February 2021). Most (but surprisingly not all) papers reported the sex of rodent subjects, although few included both sexes (27.8%) the majority were male-only studies (2.6 : 1 records). In the many studies that performed interventions or measured outcomes from the BNST area, the majority did not specify a particular subregion and analysed the structure taken as a whole (see Figure 1). On examination of the variety of terms used to describe subregions, what becomes apparent is the need for precise reporting of anatomical sites of measures in a consistent manner in order to compare across sexes, species and studies.

## Rodent models of anxiety symptoms

**Figure 2.**
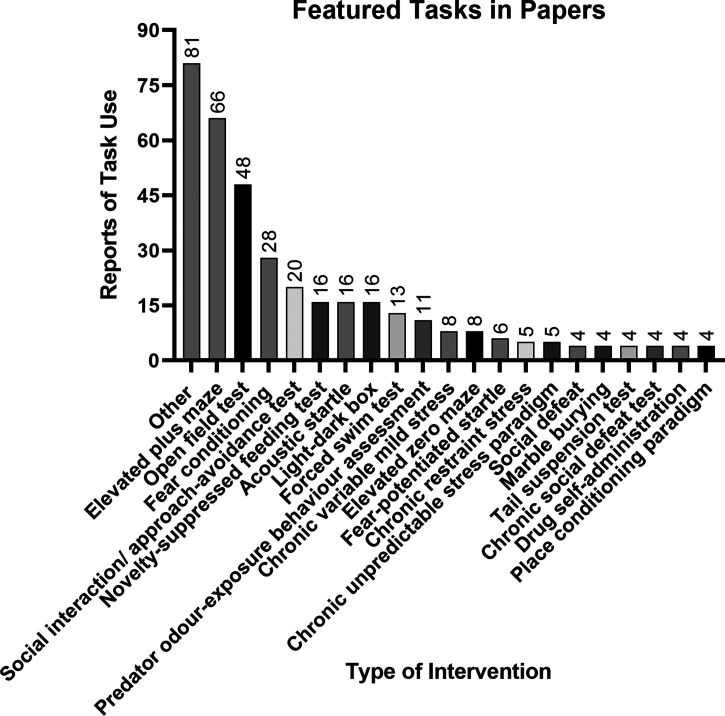
Analysis of recent literature reports of behavioural task used to elucidate BNST function in anxiety-related behaviour. Note that some papers utilised multiple tasks, each of which has been given a count of one. ‘Other’ interventions include those task variants used fewer than four times across the entirety of the literature. Above the bars are the absolute number of uses of each intervention across the referenced literature.

The validation of a task for translational potential is complex, including for pharmacological predictive validity for complex neuropsychiatric disorders like anxiety [34–36]. Another evident impression gained from the systematic review of the recent literature was that many distinct tasks were used to investigate BNST function in anxiety-like behaviour (Figure 2). The majority of the rodent studies featured as primary tasks were those that are based on innate or untrained experience of aversive (bright or open) space, i.e. the elevated plus maze (17.8% of reports) or open field maze task (12.9%). The next largest categories were tasks that use aversive associative experience i.e. Pavlovian threat/fear conditioning tasks. Below we expand on the role individual experience may play in the sensitivity and utility of these favoured tasks to model what they refer to as anxiety-like behaviour.

## Tasks evoking innate avoidance

For over a century, scientists have used mazes to study rodents’ behaviours. Mazes provide key insights into both innate and learned behaviours, as they show how a rodent instinctively navigates through a space, and additionally aids in an understanding of the mechanisms behind learning and memory. One of the first rodent mazes detailed by Willard Small in 1901 was rooted in a desire to understand Darwinian-inspired innate behaviours, and ‘to make observations upon the free expression of the animal's mental processes' [37].

The Elevated Plus Maze (EPM), pioneered by Pellow and colleagues [38], has been claimed to measure anxiety-like agoraphobia [39]. The EPM consists of two enclosed arms and two open arms arranged in a plus shape, and is commonly used to assess anxiolytic drugs to measure their efficacy (for review, see [40]). Similar to the EPM is the elevated T-maze, in which a rodent can explore two open arms and one enclosed arm [41]. These mazes are similarly useful as researchers surmise that anxiety-like behaviours can be induced by height, exposure to a new environment, and open spaces [42]. Generally, rodents tend to avoid open arms that could be dangerous for them, and exploration of these open arms is argued to be associated with lower levels of anxiety.

Another common and ethologically relevant test is the Open Field Maze (OFM), pioneered by Hall [43]. It is particularly useful as it is quite straightforward in its design; it consists of a wall-enclosed area that an animal cannot escape from. Laboratory rodents are innately averse to unknown, open, and illuminated environments, so when they are placed in the maze, their amount of locomotion and exploration is measured. Additional behaviours such as thigmotaxis (wall-hugging behaviour) and rearing can be measured to further indicate levels of anxiety-like behaviour [44].

While mazes offer some advantages over operant behavioural testing as the rodents perform more natural behaviours, there exist numerous limitations and challenges [35,45]. A potential factor for consideration is that rodents have a natural proclivity to explore [46]. As the environment is novel it should trigger an innate motivation to explore for resources or potential escape. However, with repeated testing of an experienced animal the memory of the context could influence responding read as anxiety-like behaviour [47]. The novelty-suppressed feeding test (NSF) measures another form of competing motivation, the hunger for a feeding opportunity against hyponeophagia, the neophobia induced by a new environment, which supresses an animal's feeding behaviour. The latency for food intake in a novel environment after being food-deprived is measured (for review, see [48]). Notably, the NSF test is affected by anxiolytic drugs such as barbiturates and benzodiazepines as these have been shown to decrease hyponeophagia [46].

## Tasks with acquired defensive responses

Although mazes provide one aspect of studying ethological measures, in order to fully understand the development of anxiety-like behaviour it is useful to study acquired aversions [49]. In so-called fear/threat conditioning, an animal learns to directly associate a neutral stimulus with an aversive unconditioned stimulus (US) and the conditioned response is measured [50]. In rodents, the most common fear conditioning protocols involve an electric foot shock paired with different sensory modalities including contextual, auditory, or visual cues [36,51–54]. Notably, olfaction is the key sensory modality used by rodents for identification of other animals, foraging, reproduction and social interaction [54,55] but relatively fewer studies use this modality. Olfactory fear conditioning uses an initially neutral odour (the Conditioned Stimulus, CS) paired with a foot shock (the US), and multiple studies have revealed the remarkable strength of olfactory conditioning over other senses even with the same intensity of aversive stimulus [56]. During foot-shock conditioning, the sensory information regarding the modality specific stimulus and unpleasant sensation are thought to be supported by the amygdala nuclei [57,58], which notably have reciprocal connections with the BNST [27,56]. This is in contrast with how innate predator odours have a dependence on the BNST but not the amygdala [59].

These type of classical conditioning protocols are used to typically measure conditioned freezing [60]. This has sometimes led to perhaps an over simplification of freezing behaviour to be equated with a fear-like response and the avoidance behaviour seen in mazes to be reported as simplified to an anxiety-like response. Others have discussed the defence behaviour repertoire of rodents in terms of active and passive responding [61,62]. However, given the expanding literature demonstrating the flexibility of rodent responses that are appropriate to the physical and psychological (predictability) nature of the threat cue and environment, it is unlikely that one form of behaviour is faithful to one emotional state or mood.

## Individual differences: social influences on anxiety-like behaviour

In rodents, a variety of aversive tasks, mazes or associative training, can evoke overlapping behavioural responses including avoidance, elevated heart rate, freezing, hypoactivity, suppressed food consumption, and increased vigilance [90]. In addition, laboratory rodents emit ultrasonic vocalisations (USVs, [63,64]). In rats, calls characterised at frequencies of ∼22 kHz are associated with aversive experience [65]. Measurements of USVs have been used as a correlate of stress and anxiety, which corroborates with the fact they can be reduced or blocked entirely by anxiogenic drugs [66,67]. Additionally, inter-individual variability in 22 kHz calls has commonly been reported. However, further analysis and insight into this variability has been lacking as some studies report an initial screening test to select only the animals that call (for review, see [68]). A study conducted by Brunelli ([69]) selectively bred rats on the basis extreme rates of USVs, and found rats in the genetic line with higher USVs were consistent with an altered affective phenotype such as vocalisations to touch in a new environment and low performance in a forced swim test; additionally, higher USV rates in infancy related to increased heart rate reactivity in adulthood [69]. Another study that selectively bred rats based on USV rates in infancy found that third generation rats from the low USV line spent more time in the open arms of the EPM [70]. Borta et al. ([71]) reported that vocalisation after foot-shock conditioning was more likely in rats who had spent more time in the closed arms of the EPM. However, they found that rats did not vocalise when they were in the EPM, which supports that observation that 22 kHz vocalisation is closely tied to conditioned freezing behaviour [71].

USVs may not be simply reflexive alarm calls, as rats seem to use them for communication as well, with 22 kHz calls typically being admitted in aversive situations and 55 kHz calls being admitted during appetitive situations [68]. Demaestri et al. ([72]) found that USV playback of aversive 22 kHz calls activated distinct patterns of cFos in the BNST and increased the rats’ avoidance behaviour in an elevated maze [72]. Thus, hearing USVs is also capable of inducing defensive behavioural changes in rats.

Rodents are social animals, and direct exposure to a threat is not the only way for an animal to learn and escape from danger. Thus, fear learning often occurs within a social context as an individual observes fear in other individuals. Social fear learning (SFL), otherwise known as vicarious fear learning, is a phenomenon in which an individual learns transmission of threat information through observation of a conspecific [73]. In rodents, pups can learn threat responses from their mothers as soon as they are born through SFL [74]. Later in life, mice can be fear conditioned through observation of a conspecific; Jeon et al. ([75]) showed that mice acquired fear vicariously through observing other mice reacting to aversive stimuli. An observer mouse that watched a demonstrator mouse receive foot shocks then displayed freezing behaviour as it watched the demonstrator react to foot shocks. When the demonstrator mouse was a sibling of the observer mouse, the observers exhibited increased freezing relative to observers unrelated to the conspecific. Thus, there are multiple factors at play regarding social fear learning in rodents, as the results of the relatedness between conspecifics imply that there is more than only emotional contagion involved in observational learning [75].

When housed together, rodents naturally form inter-individual hierarchies, which are important to consider when studying behavioural responses to potential threats. As early as 1938, scientists have observed and documented the presence of different social hierarchies in male mice [76]. Social dominance is defined as the animals in a social group that are most able to achieve a desirable goal, such as food or sex [77]. Evidence for variability in behaviour due to dominance hierarchies has widely increased [78], and thus, for many behavioural experiments, it is first helpful to gain an understanding of the social hierarchies between animals that are co-housed, including dominance hierarchies. In order to observe social dominance, scientists place rodents in situations that typically have predictable behavioural outcomes; for example, the submissive rodent may retreat while the dominant rodent will chase [79]. Multiple assays are employed to measure social dominance, such as a tube-test, resource competition task, or urine marking assay [80].

Recent studies have underscored differences between the anxiety-like behaviour of dominant and submissive cage-mates in both mice and rats. A meta-analysis and systematic review of the relationship between social dominance status and common behavioural phenotypes in male laboratory mice found little evidence for systematic phenotypic differences between dominant and submissive male mice [81]. Nonetheless, differential gene expression between dominant and submissive mice has been reported, yet social dominance was stable across time and not related to basal differences in mood, stress, or other physical features [82]. In contrast, submissive but not dominant mice were predisposed to anhedonia after chronic stress from restraint, tail suspension, and rat exposure [83]. Another study showed that the male offspring of dominant or submissive female mice performed differently in the EPM if they their respective dam had been exposed to prenatal restraint stress. Stressed submissive dam offspring showed the least exploration of the open arms [84]. However, it was also demonstrated that mice exposed to a chronic stress regime exhibited hyperlocomotion that was increased by triggers such as light intensity during experiments, which could impact measures of arm exploration in elevated mazes [85]. Indeed in the open field, submissive mice were reported to travel a further distance than dominant mice [78], which was regardless of sex; this could be due to hyperlocomotion from such aforementioned triggers. The same study found that social dominance accounted for phenotypic variation, but cage-identity barely accounted for any variation. Although these findings are consistent with other findings implicating social dominance's role in forming phenotypic traits in individual mice, they contradict with the findings implying the importance of cage-identity, pointing to common intra-cage variation [78]. This adds to an already mixed literature regarding social dominance and exploration. In rats, the importance of intra-cage dominant relationships was directly examined for USVs [86] and for fear conditioning by proxy; in which an animal learns from a conspecific to fear a threat cue by direct contact [87]. Submissive rats learned from dominant rats that a cue was threatening, as they displayed increased freezing to the cue. The authors found that the behaviours between cage mates as well as social dominance hierarchies in rats were predictive of social fear transmission.

## Conclusion and perspectives

It should be noted that we have discussed literature from both mice and rats herein, and there are noted differences in their behaviour and biology that is related to anxiety [88]. Moreover, the majority of studies continue to focus on readouts from male animals. The social housing and recent experience of rodents can be important influencing factors to consider. Therefore, careful attention must be taken before generalisation of findings across species, strain and sex.

Mazes that measure exploration of novel aversive contexts continue to be widely utilised as a valid approach to assess behaviour akin to anxiety despite certain limitations [35]. It is worth noting that even tasks that might superficially appear to measure similar behaviours, like the EPM and the OFM, do not necessarily always reveal the same trends of effects from interventions [35,89]. Encouragingly, more studies now characterise behaviour across multiple tasks, which may provide a clearer picture of a more ethological behaviour profile.

From a recent analysis of the literature, it becomes clear that the number of reports of interventions targeting the BNST to modulate anxiety-related behaviour is growing. Given the variability in reporting of sites of manipulation or regions where activity is correlated to behaviour, there is a need for consensus on how to refine the reporting of neuroanatomical locations. Despite these technical difficulties in comparison across studies, the BNST has certainly come more to the forefront as a target region for investigation in anxiety research [25].

## Summary

- Neuroanatomical evidence ties the BNST to anxiety-driven responses, but the role of distinct subregions remains unclear.
- Conceptions of how to experimentally define anxiety-like behaviour have evolved over time and many different tasks are used in rodents.
- Performance in typical tasks to measure ‘anxiety’ like behaviour may be sensitive to individual and social experience of the animal.

## Open Access

Open access for this article was enabled by the participation of University of Bristol in an all-inclusive *Read & Publish* agreement with Portland Press and the Biochemical Society under a transformative agreement with JISC.

## Abbreviations

BNST: bed nuclei of the stria terminalis
EPM: elevated plus maze
NSF: novelty-suppressed feeding test
OFM: open field maze
SFL: social fear learning
US: unconditioned stimulus
USVs: ultrasonic vocalisations
