# Supplementary figures and images for: The bed nucleus of the stria terminalis in threat detection: task choice and rodent experience
Source: Emerg Top Life Sci. 2022 Nov 23;6(5):457–66. doi: 10.1042/ETLS20220002 (PMC9788396; doi:10.1042/ETLS20220002)

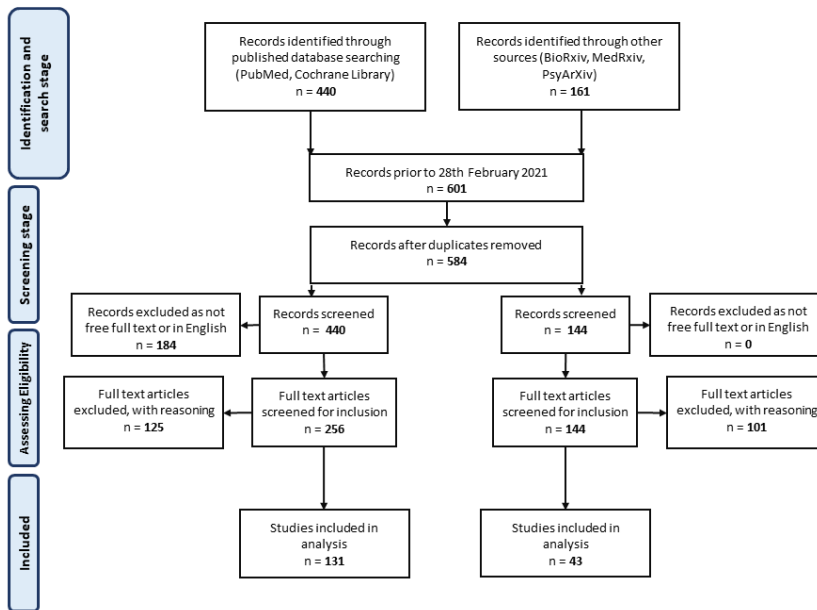

Supplement: Supplementary Material [file ETLS-6-457-s1.pdf]
